# Prediction and prognostic role of left ventricular systolic dysfunction in family screening for dilated cardiomyopathy and non‐dilated left ventricular cardiomyopathy

**DOI:** 10.1002/ejhf.3657

**Published:** 2025-04-13

**Authors:** Eva Del Mestre, Alessia Paldino, Carola Pio Loco Detto Gava, Ilaria Gandin, Marta Gigli, Davide Stolfo, Martina Setti, Giovanni Maria Severini, Beatrice Spedicati, Stefania Lenarduzzi, Giorgia Girotto, Alessandro Folgheraiter, Jacopo Giulio Rizzi, Renata Korcova, Luisa Mestroni, Marco Merlo, Matteo Dal Ferro, Gianfranco Sinagra

**Affiliations:** ^1^ Cardiovascular Department Azienda Sanitaria Universitaria Giuliano Isontina (ASUGI), University of Trieste Trieste Italy; ^2^ European Reference Network for rare, low‐prevalence, or complex diseases of the Heart (ERN GUARD‐Heart); ^3^ Biostatistics Unit, Department of Medicine Surgery and Health Science, University of Trieste Trieste Italy; ^4^ Department of Cardiology, Cardio‐Thoracic Department University Hospital of Verona Verona Italy; ^5^ Institute for Maternal and Child Health – IRCCS ‘Burlo Garofolo’ Trieste Italy; ^6^ Department of Medicine, Surgery and Health Sciences University of Trieste Trieste Italy; ^7^ Molecular Genetics Cardiovascular Institute, University of Colorado Anschutz Medical Campus Aurora CO USA

**Keywords:** Family screening, Relatives, Genetics, Dilated cardiomyopathy, Non‐dilated left ventricular cardiomyopathy

## Abstract

**Aims:**

The prognostic significance of detecting left ventricular (LV) systolic dysfunction during family screening programmes (FSPs) in relatives of probands affected by dilated (DCM) and non‐dilated left ventricular (NDLVC) cardiomyopathies remain unclear. This study sought to evaluate the prognostic role of LV systolic dysfunction detection in relatives of DCM/NDLVC probands and to define the most accurate FSP.

**Methods and results:**

Baseline and follow‐up data of first‐degree relatives of probands affected by DCM/NDLVC were collected. The primary outcome was all‐cause death and heart transplantation. Secondary heart failure (HF) and arrhythmic outcomes were also included. A total of 492 first degree relatives were enrolled. During a median follow‐up of 110 months (interquartile range 57–188 months), only subjects that previously developed LV systolic dysfunction had primary outcomes (19 vs. 0, *p* < 0.001) and secondary outcomes (HF: 12 vs. 0, *p* = 0.005; arrhythmic: 30 vs. 0, *p* < 0.001). Subjects with LV systolic dysfunction detected by FSP showed lower rate of primary outcomes (FSP: *n* = 19 [14%]; no‐FSP: *n* = 40 [37%]; *p* < 0.001) and secondary arrhythmic outcomes (FSP: *n* = 18 [13%]; no‐FSP: *n* = 41 [38%]; *p* < 0.001). In this setting, family history of arrhythmia and being carrier of a pathogenic/likely pathogenic variant are the main risk factors for LV systolic dysfunction, while LV global longitudinal strain (LV‐GLS) and Holter electrocardiogram (ECG) showed a relevant role in terms of prediction of LV systolic dysfunction and outcomes.

**Conclusions:**

Relatives of DCM/NDLVC probands who developed LV systolic dysfunction during a long follow‐up had a significant increased risk of major adverse cardiovascular outcomes. However, LV systolic dysfunction detected by FSP showed a better prognosis. In this context, genetics, Holter ECG and LV‐GLS demonstrated their functional role for disease and event prediction.

## Introduction

The genetic inheritance of dilated (DCM) and non‐dilated left ventricular (NDLVC) cardiomyopathies (CMPs) predisposes relatives of probands to develop cardiac disease.^1^ Given the complex nature of these diseases with incomplete penetrance, diagnosing CMP in relatives and defining their prognosis can be challenging, particularly when only minor signs of the disease are present.

International guidelines recommend family screening programmes (FSPs) primarily using electrocardiography (ECG) and two‐dimensional transthoracic echocardiography.^1^, ^2^ These examinations usually lead to detection of minor signs of disease, but their prognostic role in predicting clinical events in the DCM/NDLVC setting remains unclear. In this view, understanding which marker represents a definitive diagnosis of the disease and which indicates a worse prognosis for relatives could be important in refining FSPs. In addition, the potential incremental value of adding Holter ECG and left ventricular (LV) global longitudinal strain (LV‐GLS) in the FSP, which have a known role in early identification of arrhythmias^3^, ^4^ and subtle contractile abnormalities,^5^ respectively, is still poorly characterized.

Given the high cost and time involved, FSPs should be able to identify relatives at higher risk of developing future events, especially aiming to significantly reduce the incidence of sudden cardiac death (SCD) events.

In the present study we sought to assess, in a large cohort of relatives of DCM/NDLVC probands, if LV systolic dysfunction development could represent a reliable marker of disease with prognostic implications in this population. Furthermore, we aimed to clarify the diagnostic and prognostic benefits derived from more extensive FSPs including Holter ECG and LV‐GLS, along with genetic diagnosis of these individuals.

## Methods

### Study population

We retrospectively enrolled all first‐degree relatives who underwent clinical evaluation at our cardiovascular department. The study encompassed relatives of probands who were affected by DCM and NDLVC.^1^ Among the latter group, only probands with LV systolic dysfunction were included. Moreover, we identified a subgroup of probands exhibiting a more arrhythmogenic profile, named as arrhythmogenic left ventricular cardiomyopathy (ALVC), as previously described^6^, ^7^ (see online supplementary material – *paragraph 1*).

Relatives of our population were identified by (i) ‘FSP’: standardized clinical evaluation, as first medical contact, and follow‐up conducted in relatives of affected probands, starting from the age of 12; (ii) ‘no‐FSP’: first medical contact and clinical follow‐up in relatives due to symptoms and/or hospitalization for an acute presentation of the disease, and/or for an incidental detection of clinical or instrumental abnormalities already present.

**Figure 1:**
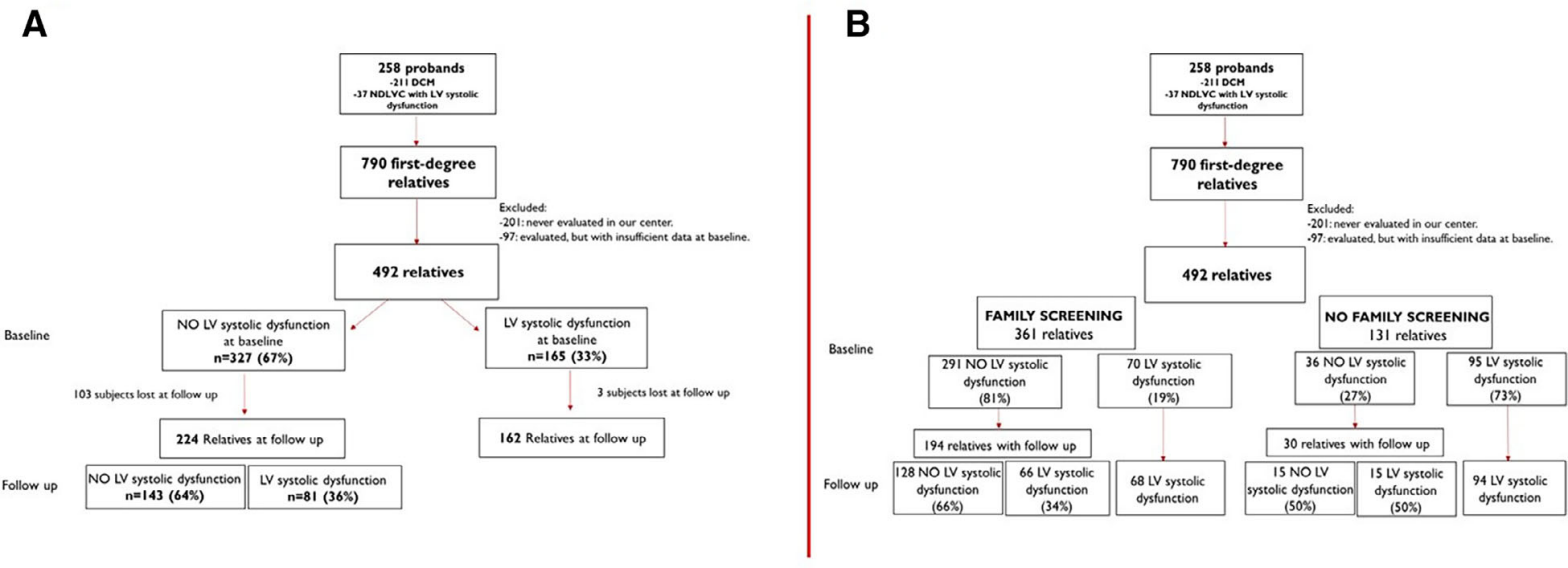
Study design. (*A*) Relatives of probands affected by dilated (DCM) and non‐dilated left ventricular (NDLVC) cardiomyopathies with left ventricular (LV) systolic dysfunction. Follow‐up data were available for 224 (69%) unaffected relatives and the median follow‐up was 130 months (interquartile range [IQR] 77–188). Among them, 81 (36%) developed LV systolic dysfunction after a median time of 53 months (IQR 23–113) versus 143 (64%) that remained unaffected during a median follow‐up of 75 months (IQR 23–139). (*B*) Family screening programme (FSP) showed a high potential detection rate of LV systolic dysfunction, both at baseline (*n* = 70 [19%]) and during follow‐up (*n* = 66 [34%]).

At baseline, we collected data on family and personal history, third‐generation family pedigree, physical examination, 12‐lead ECG, echocardiogram, Holter ECG monitoring and cardiac magnetic resonance (CMR). Relatives without baseline ECG and echocardiographic data were excluded (*Figure* *1*). Abnormalities of these exams are described in detail in online supplementary material – *paragraph 2*.

Follow‐up visits were scheduled every 1 to 3 years based on the clinical status at baseline and the results of genetic testing.^1^, ^2^ During each follow‐up, data from ECG, echocardiogram, Holter ECG monitoring and/or CMR were collected. Information about medical therapy, device implantation, and clinical outcomes was recorded.

The development of LV systolic dysfunction was represented by the detection of LV ejection fraction (LVEF) <50%, after the exclusion of secondary aetiologies (i.e. coronary artery disease).^8^, ^9^

The study was conducted in accordance with the Declaration of Helsinki and received institutional review board approval (CERU N.O. 43_2009 Em. 02 dd. 22/06/2022). Informed consent was obtained under the institutional review board policies of the hospital administration.

### Genetic testing

Probands' DNAs were analysed by next‐generation sequencing panels (see online supplementary material – *paragraph 2*), as previously reported.^10^ The pathogenicity of variants was classified into five groups, according to the American College of Medical Genetics and Genomics guidelines: pathogenetic (P), likely pathogenetic (LP), variant of uncertain significance (VUS), likely benign (LB), and benign (B).^11^

Arrhythmogenic genes were those reported by the European Society of Cardiology international guidelines for the management of CMPs.^1^

Genetic testing was offered to relatives of affected probands who were carriers of P/LP variants.

### Study clinical outcomes

The prognostic role of LV systolic dysfunction, detected during or outside a FSP, was assessed in the total population in relation to three clinical outcomes: a primary outcome comprising all‐cause death and heart transplantation; a heart failure (HF) secondary outcome including cardiovascular death, heart transplantation and ventricular assist device implantation; and an arrhythmic secondary outcome including SCD and malignant ventricular arrhythmia (defined as sustained ventricular tachycardia, ventricular fibrillation, or appropriate implantable cardioverter‐defibrillator [ICD] shocks).^12^

### Statistical analyses

Variables were expressed as median (interquartile range [IQR]) or counts (%), as appropriate. Comparisons between groups were made by the analysis of variance test on continuous variables using the Brown–Forsythe statistic when the assumption of equal variances did not hold or the non‐parametric Mann–Whitney test; the chi‐square test or the Fisher exact test were calculated for discrete variables.

Kaplan–Meier curves for the primary endpoint (log‐rank test) and cumulative incidence function for the two secondary endpoints (Gray's test) were obtained. Multivariate analyses were performed using Cox regression models with cause‐specific hazard function estimation. Martingale residuals plots were used to visually assess linearity.

Since LV‐GLS showed non‐linearity, it was included in the model using cubic splines. When dichotomization of LV‐GLS was required, the cut‐off was identified as the value corresponding to a hazard ratio (HR) of 1. To evaluate the role of clinical parameters in the prediction of LV systolic dysfunction risk, we assessed the performance of nested models using the likelihood ratio test and comparing Harrell's C‐index.

A *p*‐value<0.05 was considered statistically significant. All analyses were performed with SPSS, version 23.0 (SPSS Inc., Armonk, NY, USA) and R version 4.1 (R Foundation for Statistical Computing, Vienna, Austria).

## Results

### Left ventricular systolic dysfunction at baseline and during follow‐up

A total of 492 first‐degree relatives, out of an estimated 790 individuals derived from pedigree data across 258 families, were enrolled in the study, with the majority (73%) recruited through FSP (*Figure* *1*); 43% were carriers of a P/LP genetic variant (*Table* *1*). At baseline, 165 relatives (33%) presented LV systolic dysfunction, with a median age of 41 years (IQR 34–58), while 327 (67%) were unaffected (median age 31 years [IQR 20–43]) (*Figure* *1A*).

**Table 1:** Baseline characteristics of the study population

	Total (*n* = 492)	No LV systolic dysfunction (*n* = 327, 67)	LV systolic dysfunction (*n* = 165, 33)	*p*‐value
Age at visit (years)	38 (22–46)	31 (20–43)	41 (34–58)	0.060
Male sex	279 (57)	167 (51)	112 (68)	**<0.001**
From screening	361 (73)	291 (89)	70 (42)	**<0.001**
Familial DCM	370 (73)	245 (75)	125 (76)	0.253
P/LP variant	213 (43)	116 (35)	97 (59)	**<0.001**
Arrhythmogenic genes^a^	73 (17)	45 (16)	28 (18)	0.350
Genetics of the family				
Carrier of P/LP family variant	213 (43)	116 (35)	97 (59)	**<0.001**
Non‐carrier of P/LP family variant	64 (13)	61 (19)	3 (2)	
VUS family variant or negative	109 (22)	89 (27)	20 (12)	
Unknown	106 (22)	61 (19)	45 (27)	
Hypertension	103 (21)	40 (12)	63 (38)	**<0.001**
Hypercholesterolaemia	93 (19)	41 (12)	52 (31)	**<0.001**
Diabetes	25 (5)	7 (2)	18 (11)	**<0.001**
Alcohol	18 (4)	5 (1)	13 (8)	**0.001**
Smoking	109 (22)	59 (18)	50 (30)	**0.002**
Sport (competitive)	61 (12)	45 (14)	16 (10)	0.097
Sport (high‐intensity recreational exercise)	114 (23)	75 (23)	39 (24)	0.098
Family history of SCD	100 (20)	65 (20)	35 (21)	0.917
History of syncope	25 (5)	17 (5)	8 (5)	0.517
Previous myocarditis	12 (2)	5 (2)	7 (4)	0.071
Chemotherapy	2 (0.4)	1 (0.3)	1 (0.6)	0.633
Coronary artery disease	6 (1)	2 (0.6)	5 (3)	0.123
Pregnancy	54/213 (25)	30/160 (19)	24/53 (45)	**0.001**
NYHA class I	404 (82)	324 (99)	80 (48)	**<0.001**
ECG abnormalities	142 (29)	41 (12)	101 (61)	**<0.001**
Holter ECG abnormalities	157 (43)	49 (21)	108 (70)	**<0.001**
Missing	100 (20)	90 (27)	10 (6)	
Echo abnormalities	213 (43)	50 (15)	165 (100)	**<0.001**
LVEF (%)	50 ± 14	59 ± 6	33 ± 10	**<0.001**
LV‐GLS (%)	−16 ± 5	−18 ± 4	−11 ± 4	**<0.001**

Values are expressed as median (interquartile range), or *n* (%).

DCM, dilated cardiomyopathy; DSP, desmoplakin; ECG, electrocardiogram; FLNC, filamin C; GLS, global longitudinal strain; LMNA, lamin A; LV, left ventricular; LVEF, left ventricular ejection fraction; NYHA, New York Heart Association; PKP2, plakophilin‐2; PLN, phospholamban; P/LP, pathogenic/likely pathogenic; RBM20, RNA binding motif protein 20; SCD, sudden cardiac death; SCN5A, sodium voltage‐gated channel alpha subunit 5; TMEM43, transmembrane protein 43; VUS, variant of uncertain significance.

^a^ Arrhythmogenic genes considered are LMNA, FLNC, TMEM43, PLN, DSP, RBM20, PKP2 and SCN5A.^1^

The median follow‐up period was 110 (IQR 57–188) months: among unaffected relatives with follow‐up data available (*n* = 224), 81 (36%) developed LV systolic dysfunction, after a median follow‐up of 53 months (IQR 23–113), at the median age of 39 years (IQR 27–49) (online supplementary *Table* *S1*) and with a 1‐ and 3‐year probability of LV systolic dysfunction development of 5% (95% confidence interval [CI] 2–8%) and 16% (95% CI 11–21%), respectively (online supplementary *Figure* *S1*).

The incidence rate of LV systolic dysfunction development in this group was 39.4 × 1000 person‐years (average rate of 3.9% per person‐year).

Genetic information is reported in online supplementary material – *paragraph 3*, *Tables* *S2* and *S3*, *Figure* *S2*.

### Prognostic role of left ventricular systolic dysfunction

In the whole population during the entire follow‐up, we observed 58 (15%) primary outcome events, 38 (10%) HF and 59 (15%) arrhythmic secondary outcome events. All these events occurred only after LV systolic dysfunction development (online supplementary *Tables* *S4* and *S5*).

However, at multivariable analysis for outcomes, LVEF at baseline did not stand out as a significant predictor (*Table* *2*). Conversely, baseline lower LV‐GLS values were significantly associated with all outcomes (primary outcome: HR 1.41; 95% CI 1.25–1.58; HF secondary outcome: HR 1.47; 95% CI 1.27–1.70; arrhythmic secondary outcome: HR 1.20; 95% CI 1.10–1.31).

**Table 2:** Multivariable analysis for prediction of primary and secondary outcomes in relatives

	HR	95% CI	*p*‐value
Primary outcome (all‐cause death, HT)			
Age at first visit	1.00	0.98–1.02	0.901
Male sex	0.66	0.30–1.42	0.286
LVEF at baseline	1.02	0.99–1.06	0.135
LV‐GLS at baseline	1.41	1.25–1.58	**<0.001**
Holter ECG abnormalities	0.81	0.35–1.87	0.613
P/LP variant	0.69	0.31–1.52	0.360
HF secondary outcome (CV death, HT, VAD)			
Age at first visit	1.00	0.97–1.03	0.938
Male sex	0.47	0.19–1.12	0.089
LVEF at baseline	1.04	0.98–1.08	0.072
LV‐GLS at baseline	1.47	1.27–1.70	**<0.001**
Holter ECG abnormalities	0.87	0.30–2.52	0.801
P/LP variant	0.86	0.34–2.31	0.802
Arrhythmic secondary outcome (SCD and MVA)			
Age at first visit	1.02	0.99–1.04	0.152
Male sex	1.14	0.56–2.31	0.724
LVEF at baseline	0.99	0.97–1.03	0.833
LV‐GLS at baseline	1.20	1.10–1.31	**<0.001**
Holter ECG abnormalities	4.23	1.25–14.29	**0.020**
P/LP variant	1.33	0.65–2.71	0.435

CI, confidence interval; CV, cardiovascular; ECG, electrocardiogram; GLS, global longitudinal strain; HF, heart failure; HR, hazard ratio; HT, heart transplantation; LV, left ventricular; LVEF, left ventricular ejection fraction; MVA, malignant ventricular arrhythmia; P/LP, pathogenic/likely pathogenic; SCD, sudden cardiac death; VAD, ventricular assist device.

A LV‐GLS of −17% resulted in an appropriate cut‐off value to identify the group at higher risk of adverse events (online supplementary *Figure* *S3*). In the subgroup of relatives with LV systolic dysfunction at baseline, the LV‐GLS cut‐off value with prognostic association was lower (LV‐GLS −13%) (online supplementary *Figure* *S4*).

Finally, Holter ECG abnormalities were strongly associated with arrhythmic outcomes (HR 4.23; 95% CI 1.25–14.29) (*Table* *2*).

### Prognostic role of left ventricular systolic dysfunction detected by family screening programme

Family screening programme showed a high potential detection rate of LV systolic dysfunction, both at baseline (*n* = 70 [19%]) and during follow‐up (*n* = 66 [34%]) (*Figure* *1B*). Relatives with LV systolic dysfunction (either present at baseline or developed) detected by FSP were different from relatives in whom LV systolic dysfunction was detected mainly for symptoms (no‐FSP): they showed an earlier disease stage, with higher LVEF values, less remodelled LV and fewer ECG and Holter ECG abnormalities at first evaluation compared to those “no‐FSP” (online supplementary *Table* *S6*).

**Figure 2:**
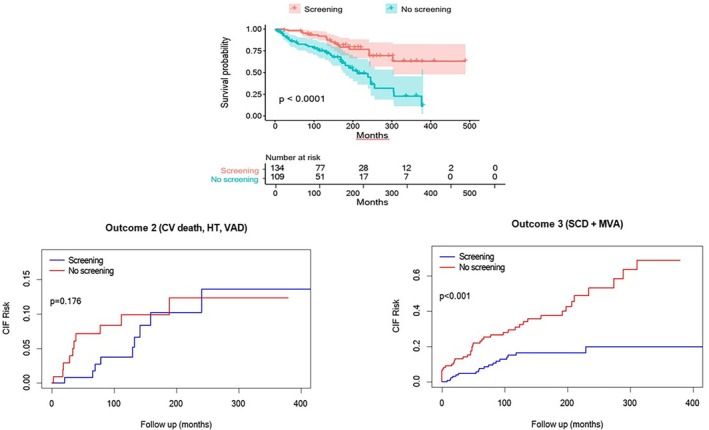
Kaplan–Meier curve for the primary outcome and cumulative incidence function (CIF) for secondary outcomes of left ventricular systolic dysfunction detected by family screening programme (FSP) or not. Relatives with left ventricular systolic dysfunction diagnosed by FSP showed significantly fewer primary outcomes (all‐cause death and heart transplantation [HT]) and secondary arrhythmic outcomes (sudden cardiac death [SCD] and malignant ventricular arrhythmia [MVA]) compared to those not diagnosed by FSP. No differences were found in secondary heart failure outcomes (cardiovascular [CV] death, HT, and ventricular assist device [VAD] implantation).

Accordingly, when comparing prognosis, survival analyses of subjects with LV systolic dysfunction showed a lower risk for the primary outcome for those relatives detected by FSP (FSP: *n* = 19 [14%]; no‐FSP: *n* = 40 [37%]; *p* < 0.001). Notably, this was driven mostly by less secondary arrhythmic outcomes (FSP: *n* = 18 [13%]; no‐FSP: *n* = 41 [38%]; *p* < 0.001), whereas HF outcomes were comparable (FSP: *n* = 9 [7%]; no‐FSP: *n* = 10 [9%]; *p* = 0.176) (*Table* *3*, *Figure* *2*).

**Table 3:** Comparison of clinical outcomes in relatives with left ventricular systolic dysfunction included versus not included in family screening

Outcomes	LV systolic dysfunction			*p*‐value
	Total (*n* = 243)	Detected by FSP (*n* = 134, 55%)	Not detected by FSP (*n* = 109, 45%)	
Death	33 (14)	10 (7)	23 (21)	**0.002**
CV death	14 (6)	6 (4)	8 (7)	0.341
SCD	8 (3)	1 (1)	7 (6)	**0.016**
HT	27 (11)	9 (7)	18 (16)	**0.016**
VAD	1 (0.4)	1 (0.7)	0 (0)	0.366
HF hospitalization	74 (30)	27 (20)	47 (43)	**<0.001**
AF	44 (18)	18 (13)	24 (22)	**0.048**
NSVT	105 (43)	45 (34)	60 (55)	**0.004**
SVT/VF	52 (21)	17 (13)	35 (32)	**<0.001**
ICD implantation	91 (37)	40 (30)	51 (47)	**0.007**
CRT‐D implantation	31 (13)	16 (12)	15 (14)	0.672
ICD shock	43 (18)	16 (12)	27 (25)	**0.009**
Primary endpoint (all‐cause death/HT)	59 (24)	19 (14)	40 (37)	**<0.001** *
Secondary HF endpoint (CV death, HT, VAD)	19 (8)	9 (7)	10 (9)	0.176**
Secondary arrhythmic endpoint (SCD, MVA)	59 (24)	18 (13)	41 (38)	**<0.001** **

Values are expressed as *n* (%).

AF, atrial fibrillation; CRT‐D, cardiac resynchronization therapy‐defibrillator; CV, cardiovascular; FSP, family screening programme; HF, heart failure; HT, heart transplantation; ICD, implantable cardioverter‐defibrillator; LV, left ventricular; MVA, malignant ventricular arrhythmia; NSVT, non‐sustained ventricular tachycardia; SCD, sudden cardiac death; SVT, sustained ventricular tachycardia; VAD, ventricular assist device; VF, ventricular fibrillation.

* Kaplan–Meier curves and log‐rank test.

** Cumulative incidence function analysis.

### Predictors for left ventricular systolic dysfunction development during follow‐up

Since LV systolic dysfunction was highly associated with development of outcomes, we analysed risk factors associated with LV systolic dysfunction development, and which tests included in FSP were predictive of LV systolic dysfunction development, in our study population.

#### Risk factors associated with left ventricular systolic dysfunction development

At multivariate analysis, only being carrier of a P/LP variant (HR 1.96; 95% CI 1.12–3.43; *p* = 0.0 19) and presenting a family history of ALVC (HR 3.44; 95% CI 1.84–6.45; *p* < 0.001) or SCD (HR 1.73; 95% CI 1.04–2.89; *p* = 0.037) emerged significantly associated with the development of LV systolic dysfunction (*Table* *4*, *Table* *S7*). Of note, in our cohort, acquired factors (hypertension, obesity, diabetes) were not associated with this phenotypic outcome.

**Table 4:** Univariable and multivariable analysis for development of left ventricular systolic dysfunction (from unaffected relatives at baseline)

Cox regression	Univariable			Multivariable		
	HR	95% CI	*p*‐value	HR	95% CI	*p*‐value
Sex	1.13	0.84–2.12	0.221			
Age	1.01	0.99–1.02	0.297			
P/LP variant	1.86	1.14–3.03	**0.013**	1.96	1.12–3.43	**0.019**
Arrhythmogenic genes^a^	1.65	1.00–2.73	0.050	0.81	0.43–1.53	0.513
Family history of ALVC	1.23	1.13–1.40	**<0.001**	3.44	1.84–6.45	**<0.001**
Hypertension	1.150	1.00–2.06	0.048	0.62	0.29–1.32	0.211
Hypercholesterolaemia	1.83	1.06–3.16	0.030	1.54	0.79–3.01	0.207
Diabetes	1.32	0.32–5.45	0.067			
Alcohol assumption	0.88	0.12–6.39	0.904			
Smoking	1.51	0.97–2.50	0.106			
Sports (competitive or high‐intensity recreational exercise)	1.40	0.87–2.26	0.161			
Family history of SCD	2.32	1.49–3.61	**< 0.001**	1.73	1.04–2.89	**0.037**
Previous myocarditis	0.53	0.07–3.84	0.0531			
Coronary artery disease	1.93	0.27–13.94	0.516			
Pregnancy	1.91	0.94–3.89	0.073			

ALVC, arrhythmogenic left ventricular cardiomyopathy; CI, confidence interval; DSP, desmoplakin; FLNC, filamin C; HR, hazard ratio; LMNA, lamin A; P/LP, pathogenic/likely pathogenic; PLN, phospholamban; PKP2, plakophilin‐2; RBM20, RNA binding motif protein 20; SCD, sudden cardiac death; SCN5A, sodium voltage‐gated channel alpha subunit 5; TMEM43, transmembrane protein 43.

^a^ Arrhythmogenic genes considered are LMNA, FLNC, TMEM43, PLN, DSP, RBM20, PKP2 and SCN5A.^1^

#### Tests included in family screening programme predictive of left ventricular systolic dysfunction development

At baseline evaluation, multiple subtle instrumental abnormalities were detected in relatives despite LVEF above normal values (online supplementary *Table* *S8*). To investigate the predictive value of ECG, echocardiography, Holter ECG abnormalities and LV‐GLS at baseline, several multivariate models were compared. When using only ECG and echocardiography, we obtained poor discrimination (C‐index ECG + Echo: 0.65), whereas good results were obtained by adding Holter ECG (C‐index ECG + Echo + Holter ECG: 0.77, *p* < 0.001). The best performance was achieved with the additional inclusion of LV‐GLS (C‐index ECG + Echo + Holter ECG + LV‐GLS: 0.78, *p* = 0.011) (online supplementary *Table* *S9*).

Based on the cubic spline analysis, −18% of LV‐GLS appeared as an appropriate cut‐off for dichotomization (online supplementary *Figure* *S5*). The analysis, including LV‐GLS as a dichotomous variable, confirmed all results.

Based on the 1‐year and 3‐year probabilities of LV systolic dysfunction development in this cohort, the only factors capable of determining the appropriate follow‐up interval from 1 to 3 years (as suggested by the European guidelines^1^) were: a family history of ALVC (21% risk of LV systolic dysfunction development at 1 year), a family history of SCD (11% risk of LV systolic dysfunction development at 1 year), and abnormal LV‐GLS (11% risk of LV systolic dysfunction development at 1 year) (online supplementary *Figure* *S1*). The presence of at least one of these factors supports a follow‐up interval of 1 year.

## Discussion

### Main findings

The primary objective of this study was to characterize one of the largest population of relatives of probands affected by DCM and NDLVC with LV systolic dysfunction, evaluating the prognostic value of LV systolic dysfunction detection. The key findings can be summarized as follows: (i) a significant prevalence of LV systolic dysfunction was found in our study population, with a median age of 40 years for disease onset. Relatives who remained unaffected (i.e. without LV systolic dysfunction) throughout the follow‐up period did not experience any clinical event, indicating a favourable prognosis; (ii) LV systolic dysfunction detected by FSP showed a significant better prognosis; and (iii) independently of LVEF at baseline, the inclusion of Holter ECG and LV‐GLS, along with genetic screening, played an essential role in our FSP in terms of prediction of LV systolic dysfunction, and thus clinical outcomes.

These findings may improve cost effectiveness of FSP and the prognostic management of relatives.

### Role of left ventricular systolic dysfunction

We here describe a disease penetrance in relatives of probands affected by DCM/NDLVC with LV systolic dysfunction of 36%. This finding is significantly higher compared to previous reports on FSP, showing less than 10% penetrance,^13^, ^14^ but more similar to that in Cabrera‐Romero *et al*.^15^

These differences could be correlated to distinctive features of our study, such as the inclusion of more heterogeneous genetic background and clinical forms of the probands (NDLVC with LV systolic dysfunction and DCM) and the presence of a long follow‐up of the relatives (i.e. almost 10 years), that might explain this higher rate of disease detection.

Clinical manifestations of the disease were most likely to occur between the ages of 25–55 years, consistent with previous literature. This age range seems the most important to follow by a FSP with optimized cost‐effectiveness balance. Earlier screening should be reserved to relatives with high‐risk conditions, such as families with arrhythmogenic genes or relatives engaged in competitive sports.^1^, ^2^, ^16^, ^17^

Notably, our study shows that LV systolic dysfunction in relatives is significantly associated with the primary and secondary outcomes, and this correlation is independent of the absolute LVEF value. As a matter of fact, in our population, arrhythmic and non‐arrhythmic adverse events occurred only after LV systolic dysfunction development. These results support current international guidelines, suggesting ICD implantation in primary prevention only in relatives with LVEF <50%,^1^, ^18^ even for high‐risk subjects (i.e. arrhythmic genotype carriers).

We are aware that in probands, especially with high‐risk genotypes, arrhythmic events may precede LV systolic dysfunction; the reasons behind this difference with our study population of relatives (detected also by Cabrera‐Romero *et al*.^15^ in their population) are yet to be elucidated, but a possible protective role of some therapeutic choices (e.g. lifestyle modifications or early pharmacological treatment) during FSP may be hypothesized.

### The role of family screening programmes

Family screening represents an intensive and time‐demanding task for all the centres specialized in managing subjects affected by CMPs, involving plenty of resources and high costs.^19^ Moreover, recent studies, showing a low rate of DCM detection in large relatives' cohorts, have questioned the utility of FSP.^13^, ^14^ For these reasons, there is a growing demand for a more rational and personalized approach that allows the discharge of low‐risk family members, optimizing resources and reducing psychological stress for these subjects.^13^ On the other hand, there is still a strong international support for the beneficial role of FSP.

Previous studies demonstrated the clinical relevance of FSP in DCM, revealing that it facilitates diagnosis in the early‐stage and significantly improves the prognosis in terms of death and heart transplantation, although the potential impact on SCD and malignant ventricular arrhythmia is still not properly elucidated.^20^

Although it would be unethical to conduct a randomized controlled trial in relatives (FSP vs. no‐FSP), our retrospective study supports the evidence of a better prognosis for these individuals when followed by FSP, particularly in terms of arrhythmic events. Importantly, with respect to relatives detected mostly for symptoms, screened relatives received fewer ICD implantations and experienced fewer ICD shocks, potentially due to earlier therapeutic management.

Our results suggest that the prognostic target of FSP primarily should be the prediction of LV systolic dysfunction, since in our cohort clinical events in relatives manifest only after LV systolic dysfunction development. Thus, our analyses strongly support the necessity to continue extensive FSP and this is not only due to the relevant rate of disease development in relatives of probands affected by DCM and NDLVC but, more importantly, because of a better prognosis of these subjects. FSP remains a crucial tool for improving outcomes and should be prioritized despite the challenges it poses.

### Implementation of family screening programmes

Genetic screening has changed the clinical approach to CMPs and FSP, and nowadays it is largely applied. The large proportion of carriers of P/LP variants (43%) classifies our population at high risk of developing LV systolic dysfunction and outcomes compared to previous studies on FSP.^13^, ^14^ In fact, in our study, penetrance and incidence of LV systolic dysfunction were found to be influenced by genetic background more than environmental factors. Indeed, the detection of a P/LP variant in relatives was one of the main predictors of disease development. Although the impact of a P/LP variant is clearly significant, the risk for carriers is only twice that of other relatives (OR 1.964, 95% CI 1.123–3.431), and its presence does not strongly discriminate the need for a shorter follow‐up interval, unlike other risk factors such as a family history of ALVC, family history of SCD, or abnormal LV‐GLS. This can be attributed to the fact that 20% of LV systolic dysfunction cases are represented by relatives of probands with VUS or negative genetic testing. This finding highlights that a purely monogenic cause accounts for only a portion of these familial myocardial diseases.

Moreover, ECG and echocardiogram represent the cornerstone for an adequate FSP, as recommended by the main international guidelines.^1^, ^2^ Our results are consistent with previous reports, showing a higher prevalence at baseline of pathological ECG and echocardiogram in relatives who developed LV systolic dysfunction during follow‐up, compared to persistent unaffected family members.

Furthermore, in our study, LV‐GLS proves capable of detecting subtle abnormalities in contractile LV function and improves outcome prediction in family members with a normal LVEF, confirming previous preliminary studies.^5^, ^21^, ^22^ We found distinct cut‐off values of LV‐GLS: in unaffected relatives, a value inferior to −18% correlates with LV systolic dysfunction development; a value inferior to −17%, independently of LVEF, predicts arrhythmic and non‐arrhythmic outcomes. Confirmation in future studies is required.

Finally, the inclusion of Holter ECG significantly implements a standard FSP in predicting the development of LV systolic dysfunction in unaffected relatives at baseline and major arrhythmic events in all relatives, affected and unaffected, during follow‐up.

Despite the added value of LV‐GLS and Holter ECG in family screening, it is necessary to acknowledge the potential impact on healthcare system resources and costs. However, it is worth noting that Holter ECG is a low‐cost tool and that LV‐GLS is increasingly integrated into modern echocardiography machines and software commonly used in clinical practice, which facilitates their implementation without requiring significant additional investments. Moreover, their use enables a more accurate stratification of low‐risk individuals, allowing for the deferral of subsequent follow‐up evaluations, thus optimizing resource allocation and potentially reducing overall costs.

In conclusion, according to our result, the inclusion of LV‐GLS and Holter ECG, in addition to ECG and echocardiogram, significantly ameliorates FSP, enhancing disease prediction and prognostic assessment.

### Limitations

The study is limited by its retrospective and single registry‐based design, which may restrict the generalizability of the findings. Furthermore, the hospital's role as a tertiary referral centre for CMPs, the presence of LV systolic dysfunction at baseline and the high percentage of relatives detected by FSP may introduce selection bias in the study population and outcomes.

Although the total at‐risk population of first‐degree relatives, derived from the pedigree, was 790, we were able to achieve a good coverage rate, including 492 subjects (62%). Although we included only subjects with at least an ECG and echocardiographic evaluation, there were instances of some missing data for Holter ECG (22%), LV‐GLS (10%) and a substantial proportion of missing CMR data (almost 70%). The limited rate of CMR application in our study population still prevented us from fully exploring its predictive capacity in relation to other data.

Although our study benefits from one of the longest follow‐ups available for this type of population, for the significant variability in penetrance, late‐onset, and heterogeneous expression of the disease, further research with larger cohorts and more standardized protocols is warranted to validate and extend our findings.

## Conclusion

Relatives of probands affected by non‐hypertrophic LV CMP with LV systolic dysfunction, especially in the presence of a P/LP variant and when the proband exhibits a more arrhythmogenic phenotype, represent a high‐risk population for development of LV systolic dysfunction, typically occurring around the age of 40. LV systolic dysfunction appears to be a crucial factor before the manifestation of major cardiac events in these subjects. However, when LV systolic dysfunction is detected through FSP rather than symptom onset or occasional diagnosis, a more favourable prognosis is demonstrated. The incorporation of Holter ECG and LV‐GLS into the FSP protocol enhances its ability to identify asymptomatic relatives at higher risk of LV systolic dysfunction development or major event occurrence, enabling personalized follow‐up intervals.

## Supporting information


**Appendix S1.** Supporting Information.
