# Supplementary material for: Prediction and prognostic role of left ventricular systolic dysfunction in family screening for dilated cardiomyopathy and non‐dilated left ventricular cardiomyopathy
Source: Eur J Heart Fail. 2025 Apr 13;27(12):3260–8. doi: 10.1002/ejhf.3657 (PMC12803633; doi:10.1002/ejhf.3657)
Supplement: Supplementary file 1 — Appendix S1. Supporting Information. [file EJHF-27-3260-s001.docx]

**Prediction and Prognostic Role of Left Ventricle Systolic Disfunction in Family Screening for Dilated Cardiomyopathy and Non-Dilated Left Ventricular Cardiomyopathy**

Eva Del Mestre^1*^, MD, Alessia Paldino^1*^, MD, PhD, Carola Pio Loco detto Gava^1^, MD, Ilaria Gandin^2^, PhD, Marta Gigli^1^, MD, Davide Stolfo^1^, MD, PhD, Martina Setti^3^, MD, Giovanni Maria Severini^4^, PhD, Beatrice Spedicati^4^, MD, Stefania Lenarduzzi^4^, MS, Giorgia Girotto^4,5^, PhD, Alessandro Folgheraiter^1^, MD, Jacopo Rizzi^1^, MD, Renata Korcova^1^, MD, PhD, Luisa Mestroni^6^, MD, Marco Merlo^1^, MD, Matteo Dal Ferro^1^, MD, Gianfranco Sinagra^1^, MD

^1^ Cardiovascular Department, Azienda Sanitaria Universitaria Giuliano Isontina (ASUGI), University of Trieste, Trieste, Italy. Member of the European Reference Network for rare, low-prevalence, or complex diseases of the Heart (ERN GUARD-Heart).

^2^ Biostatistics Unit, Department of Medicine, Surgery and Health Science, University of Trieste.

^3^ Department of Cardiology, Cardio-Thoracic Department, University Hospital of Verona, Italy.

^4^ Institute for Maternal and Child Health - I.R.C.C.S. "Burlo Garofolo", Trieste, Italy.

^5^ Department of Medicine, Surgery and Health Sciences, University of Trieste, Trieste, Italy.

^6^ Molecular Genetics, Cardiovascular Institute, University of Colorado Anschutz Medical Campus, Aurora, Colorado.

* Eva Del Mestre and Alessia Paldino contributed equally to the study and shall be considered co-first authors.

**Supplementary material- paragraph 1: Arrhythmogenic Left Ventricular Cardiomyopathy**

Arrhythmogenic left ventricular cardiomyopathy was defined by the presence of more than one criterion among the following: a) resuscitated cardiac arrest, sustained ventricular tachycardia (VT), appropriate implantable cardioverter defibrillator interventions, b) unexplained syncope, c) rapid non-sustained ventricular tachycardia defined as ≥5 consecutive ventricular beats, lasting <30 seconds, with a rate ≥150/min on 24‐hour Holter monitoring, d) ≥1000 premature ventricular contractions or e) ≥50 couplets during the 24 hours.

**Supplementary material- paragraph 2: ECG, Echocardiography, ECG Holter, Cardiac Magnetic Resonance abnormalities and genetic testing**

ECG abnormalities comprehended high degree of atrioventricular (AV) blocks, left bundle branch block (LBBB), presence of pathological Q waves, negative T waves (>1mm, in 2 consecutive leads and not including aVR or V1), fragmented QRS complex and low QRS voltages.(1–3)

Echocardiography measurements were performed according to current international guidelines.(4) Echo abnormalities were defined as LV dilatation and the presence of ≥ 1 abnormal kinetic segment. Two-dimensional speckle-tracking analysis was performed on the first echocardiogram images using the latest version of the vendor independent software (2D CPA software; TomTec Imaging Systems, Unterschleissheim, Germany), as previously described.(5)

ECG Holter abnormalities comprehended premature ventricular complexes (PVC) > 1000/24 hours and non-sustained ventricular tachycardia (NSVT) ≥ 3 beats at a rate of ≥120 bpm, and a high degree of AV blocks.(1,3)

Late gadolinium enhancement presence was considered as Cardiac Magnetic Resonance abnormality.

The list of genes incorporated into the NGS panel for structural cardiomyopathies includes 36 genes: *ACTC1, DSP, MYH7, SCN5A, TTR, TCAP, BAG3, FLNC, MYL2, TNNC1, VCL, TBX20, DES, GLA, MYL3, TNNI3, LDB3, FHOD3, DMD, LAMP2, NEXN, TNNT2, NEBL, FLNA, DSC2, LMNA, PKP2, TPM1, JUP, RBM20, DSG2, MYBPC3, PLN, TTN, FHL1, PRKAG2.*

**Supplementary material- paragraph 3: Genetic diagnosis in study population and LV systolic disfunction development**

The study population was sub-classified according to the genetic diagnosis as: 213 carriers of the familial P/LP variant (group 1) (43%); 64 non-carrier of the familial P/LP variant (group 2) (13%); 109 relatives of probands with VUS or negative genetic test (group 3) (22%). Genetic data was unknow for 106 relatives (21%) and their probands.

In group 1, *TTN* (n=87, 41%), *FLNC* (n=32, 15%) and *LMNA* (n=23, 11%) were the most represented genes.

Although the majority of relatives with LV systolic disfunction belong to group 1 (78%), disease development was documented also in group 2 (2%) and group 3 (20%) (Supplementary Table 2, Supplementary Figure 1). The median age of LV systolic disfunction detection was 40 years (IQR 30-51), with no significant differences between the groups. Notably, the subjects of group 2 were free from events, while group 1 showed a significantly higher rate of arrhythmic events (20%) in comparison with group 3 (8%) (Supplementary Table 3).

In group 1, *TTN* (n=87, 41%), *FLNC* (n=32, 15%) and *LMNA* (n=23, 11%) were the most represented genes (Supplementary Figure 1). Global disease penetrance among carriers was 67% (143/213 subjects), lower in carriers of *TTN* variants (62%, 54/87 subjects; LV systolic disfunction: n=16, 18% at the baseline and n=38, 44% during follow-up; median age of 42 years IQR 35-54), and higher in carriers of *FLNC* (69%, 22/32 subjects; LV systolic disfunction: n=6, 19% at the baseline and n=16, 50% during follow-up; median age of 39 years IQR 31-44) and *LMNA* (70%, 16/23 subjects; LV systolic disfunction: n=10, 44% at the baseline and n=6, 26% during follow-up; median age of 43 years IQR 35-48) variants (Supplementary Figure 4). Carriers of P/LP variants of *MYH7,* even if they had a relatively disease-low penetrance (9/15 subjects, 60%), showed the youngest age of LV systolic disfunction diagnosis compared to the other gene groups (median age of 21 years, IQR 17-35).

|  | **Total population – with follow-up (n=386)** | **No LV systolic disfunction**  **at follow-up**  **(n=143, 37%)** | **LV systolic disfunction at follow-up**  **(n=81, 21%)** | **LV systolic disfunction at baseline (n=162, 42%)** |
| --- | --- | --- | --- | --- |
| Age baseline (years, IQR) | 38 (29-51) | 30 (18-42) | 31 (23-44) | 41 (34-58) |
| Sex (male) (%) | 227 (59%) | 66 (46%) | 51 (63%) | 110 (68%) |
| NYHA class 1 at baseline (%) | 302 (78%) | 143 (100%) | 79 (98%) | 80 (49%) |
| LVEF at baseline (%) | 58 (45-62) | 61 (57-65) | 53 (51-58) | 33 (25-42) |
| LV-GLS at baseline (%) | -14.6 (16.6-9.9) | -19.5 (21.1-18.2) | -15.5 (17.0-13.0) | -11.1 (12.6-9.9) |
| ECG abnormalities (%) | 135 (35%) | 8 (6%) | 27 (33%) | 100 (62%) |
| Holter ECG abnormalities (%) | 155 (47%) | 2 (2%) | 45 (63%) | 108 (71%) |
| P/LP variants (%) | 195 (51%) | 53 (37%) | 46 (57%) | 96 (59%) |
| P/LP variants in arrhythmic genes (%) | 64 (17%) | 15 (11%) | 21 (26%) | 28 (17%) |
| ACE-i/ARB/ARNI present or initiated at baseline (%) | 151 (39%) | 8 (6%) | 10 (12%) | 133 (82%) |
| Beta-blockers present or initiated at baseline (%) | 159 (41%) | 12 (8%) | 14 (17%) | 133 (82%) |
| MRA present or initiated at baseline (%) | 55 (14%) | 0 | 0 | 55 (34%) |
| Duration of follow-up (months, IQR) | 110 (57-188) | 130 (77-188) | | 94 (38-169) |

**Supplementary Table 1**. Baseline clinical, instrumental, and genetic features of relatives (with available follow-up) according to development of LV systolic disfunction.

ACE-i= Angiotensin-converting enzyme inhibitors; ARB= Angiotensin Receptor Blockers; ARNI= angiotensin receptor neprilysin inhibitor; EF= Ejection Fraction, GLS= Global Longitudinal Strain; HF=heart failure; LV= Left Ventricular; LV= Left Ventricle; P/LP= Pathogenetic/Likely Pathogenetic.

| Groups according to the familial  genetic background  (n=386) | **LV systolic disfunction**  **at baseline** | **LV systolic disfunction**  **during follow-up** | **Unaffected** |
| --- | --- | --- | --- |
| **Group 1**: P/LP variant in proband, LP/P variant in relative.  n=213 | 97 (46%) | 46 (22%) | 70 (33%) |
| **Group 2**: P/LP variant in proband, negative relative.  n=64 | 0 (0%) | 3 (5%) | 61 (95%) |
| **Group 3**: VUS variant or negative result in proband.  n=109 | 20 (18%) | 17 (16%) | 72 (66%) |

**Supplementary Table 2. Left ventricular systolic dysfunction according to the familial genetic background of the proband.** Relatives were divided in three groups according their familial genetic background (Group 1: P/LP variant in proband, the same P/LP variant in relative; Group 2: P/LP variant in proband, negative relative; Group 3: VUS variant or negative result in proband). Out of 183 affected relatives, the majority of them belong to the group 1 (n=143/183, 78%). Although at lower rate, LV systolic dysfunction was documented also in group 2 (n=3/183, 2%) and group 3 (n=37/183, 20%).

LV = Left Ventricle; P/LP= Pathogenetic/Likely Pathogenetic; VUS=Variant of Uncertain Significance.

|  | **Group 1**  **(195 subjects)** | **Group 2**  **(28 subjects)** | **Group 3**  **(87 subjects)** | **P value** |
| --- | --- | --- | --- | --- |
| Primary Outcomes  **(All-cause death/HT)** | 34 (17%) | 0 (0%) | 10 (12%) | 0.033 |
| Secondary HF Outcomes  **(CV death, HT, VAD)** | 23 (12%) | 0 (0%) | 6 (7 %) | 0.087 |
| Secondary Arrhythmic Outcomes  **(SCD, MVA)** | 39 (20%) | 0 (0%) | 7 (8%) | < 0.001 |

**Supplementary Table 3. Outcomes according to the genetic groups**. Group 1: P/LP variant in proband, P/LP variant in relative; Group 2: P/LP variant in proband, negative relative; Group 3: VUS variant or negative result in proband.

CV=Cardiovascular; HT=Heart Transplantation; MVA=Major Ventricular Arrhythmias; SCD=Sudden Cardiac Death; VAD=Ventricular Assist Device.

| **Outcomes** | **Total population**  **(n=386)** | **No LV systolic disfunction**  **at follow-up (n=143)** | **LV systolic disfunction at follow-up**  **(n=81)** | **LV systolic disfunction at baseline**  **(n=162)** |
| --- | --- | --- | --- | --- |
| Death (%) | 33 (9%) | 0 (0%) | 6 (7%) | 27 (17%) |
| CV death (%) | 14 (4%) | 0 (0%) | 3 (4%) | 11 (7%) |
| SCD (%) | 9 (2%) | 0 (0%) | 3 (4%) | 6 (4%) |
| HT (%) | 27 (7%) | 0 (0%) | 8 (10%) | 19 (12%) |
| VAD (%) | 1 (0.3%) | 0 (0%) | 1 (1%) | 0 (0%) |
| HF hospitalization (%) | 74 (19%) | 0 (0%) | 13 (16%) | 61 (38%) |
| AF (%) | 44 (11%) | 2 (1%) | 11 (14%) | 29 (18%) |
| NSVT (%) | 105 (27%) | 4 (3%) | 35 (43%) | 66 (41%) |
| SVT/VF (%) | 52 (13%) | 0 (0%) | 10 (12%) | 42 (26%) |
| ICD implantation (%) | 91 (24%) | 0 (0%) | 25 (31%) | 66 (41%) |
| CRT-D implantation (%) | 31 (8%) | 0 (0%) | 10 (12%) | 21 (13%) |
| ICD shock (%) | 43 (11%) | 0 (0%) | 10 (12%) | 33 (20%) |
| **Primary Outcomes (all-cause death/HT) (%)** | 58 (15%) | **0 (0%)** | 14 (17%) | 44 (27%) |
| **Secondary HF Outcomes (CV death/HT/VAD) (%)** | 38 (10%) | **0 (0%)** | 10 (12%) | 28 (17%) |
| **Secondary Arrhythmic Outcomes  (SCD, MVA) (%)** | 59 (15%) | **0 (0%)** | 12 (15%) | 47 (29%) |

**Supplementary Table 4 (Outcomes).** Clinical outcomes of the study population with available follow-up on the base of the presence or the development of LV systolic disfunction at follow up.

AF=Atrial Fibrillation; CRT-D= Cardiac Resynchronization Therapy-Defibrillator; CV= Cardiovascular; HT=Heart Transplantation; ICD=Implantable Cardioverter Defibrillator; LV = Left Ventricle; MVA=Major Ventricular Arrhythmias; NSVT=Non-sustained Ventricular Tachycardia; SCD=Sudden Cardiac Death; SVT=Sustained Ventricular Tachycardia; VF=Ventricular Fibrillation; VAD=Ventricular Assist Device.

| **PRIMARY OUTCOME (all cause death+HT)**  **(n=386)** | | | | | | |
| --- | --- | --- | --- | --- | --- | --- |
| ‍ | Free from events within 5 years  n=248 | Endpoint within 5 years  n=19 | Chi-square  (df) | p-value |  | Free from events and follow-up less than 5 years |
| LV systolic disfunction  n=155 | 136 | 19 | 12.98  (1) | <0.001 |  | 49 |
| NO LV systolic disfunction  n=112 | 112 | 0 |  |  |  | 70 |
|  | | | | | | |
| **HF SECONDARY OUTCOME (CV death+HT+VAD)**  **(n=386)** | | | | | | |
| ‍ | Free from events within 5 years  n=255 | Endpoint within 5 years  n=12 | Chi-square  (df) | p-value |  | Free from events and follow-up less than 5 years |
| LV systolic disfunction  n=148 | 136 | 12 | 7.77  (1) | 0.005 |  | 56 |
| NO LV systolic disfunction  n=112 | 112 | 0 |  |  |  | 70 |
|  | | | | | | |
| **ARRHYTHMIC SECONDARY OUTCOME (SCD+MVA)**  **(n=386)** | | | | | | |
| ‍ | Free from events within 5 years  n=248 | Endpoint within 5 years  n=19 | Chi-square  (df) | p-value |  | Free from events and follow-up less than 5 years |
| LV systolic disfunction  n=151 | 121 | 30 | 23.19  (1) | <0.001 |  | 53 |
| NO LV systolic disfunction  n=112 | 112 | 0 |  |  |  | 70 |

**Supplementary Table 5. Primary and Secondary Outcomes considering a 5-year observational window.** Outcomes only occur in relatives with LV systolic disfunction. Relatives with no events during the 5-year observational window and with a follow-up less than 5 years (last column) are excluded from the statistical analysis.

CV=Cardiovascular; HF=Heart Failure; HT=Heart Transplantation; LV= Left Ventricle; MVA=Major Ventricular Arrhythmias; SCD=Sudden Cardiac Death; VAD=Ventricular Assist Device.

|  | **LV systolic disfunction (n=243)** | **LV systolic disfunction –**  **detected by FSP**  **(n=134, 55%)** | **LV systolic disfunction –**  **not detected by FSP  (non-FSP)**  **(n=109, 45%)** | **P value** |
| --- | --- | --- | --- | --- |
| Age at first visit (years, IQR) | 39 (28-51) | 36 (25-48) | 42 (31-53) | 0.023 |
| Sex (male) (%) | 161 (66%) | 86 (64%) | 75 (69%) | 0.448 |
| LV dysfunction at baseline (%) | 162 (67%) | 68 (51%) | 94 (86%) | <0.001 |
| P/LP variant (%) | 142 (58%) | 83 (62%) | 59 (54%) | 0.300 |
| Familial DCM | 182 (75%) | 106 (79%) | 76 (70%) | 0.162 |
| Sinus Rhythm (%) | 223 (92%) | 129 (96%) | 94 (86%) | 0.005 |
| 1^st^ degree AV block (%) | 37 (15%) | 21 (16%) | 16 (15%) | 0.867 |
| LBBB (%) | 31 (13%) | 17 (13%) | 14 (13%) | 0.816 |
| Negative T waves (%) | 88 (36%) | 37 (28%) | 51 (47%) | <0.001 |
| Q waves (%) | 23 (9%) | 11 (8%) | 12 (11%) | 0.372 |
| Fragmented QRS (%) | 39 (16%) | 18 (13%) | 21 (19%) | 0.144 |
| Low voltages (%) | 26 (11%) | 9 (7%) | 17 (16%) | 0.014 |
| PVC ≥ 1000/24h (%) | 110 (45%) | 56 (42%) | 54 (49%) | 0.294 |
| PVC couplets ≥500/24h (%) | 36 (15%) | 15 (11%) | 21 (19%) | 0.037 |
| NSVT > 3 beats (%)  Missing (%) | 102 (42%) | 48 (35%) | 54 (50%) | 0.049 |
| AF (%) | 24 (10%) | 9 (7%) | 15 (14%) | 0.060 |
| LVDD (mm) | 60 (54-65) | 58 (53-62) | 63 (57-70) | <0.001 |
| LV EF at baseline (%) | 42 (28-51) | 48 (36-54) | 33 (24-44) | <0.001 |
| LV EF follow up (%) | 45 (34-49) | 46 (39-49) | 40 (27-48) | 0.013 |
| LV GLS at baselines (%) | -13.0 (9.7-16.2) | -14.4 (11.4-17.0) | -11.1 (7.4-15.1) | <0.001 |
| Mitral regurgitation (moderate-severe) (%) | 50 (21%) | 16 (12%) | 34 (31%) | <0.001 |
| RV dysfunction (%) | 47 (19%) | 17 (13%) | 30 (27%) | 0.002 |
| LGE (%) | 50 (21%) | 23 (17%) | 27 (25%) | 0.076 |
| ICD implantation (%) | 91 (37%) | 40 (30%) | 51 (47%) | 0.007 |
| Follow-up (months) | 126 (50-174) | 132 (116-149) | 117 (99-134) | 0.192 |

**Supplementary Table 6.** Baseline characteristic of the study population with LV systolic disfunction at the baseline or at follow-up according to the identification by family screening or not (Screening versus No screening).

AF=Atrial Fibrillation; AV=Atrio-Ventricular; DCM=Dilated Cardiomyopathy; EF=Ejection Fraction; GLS=Global Longitudinal Strain; ICD=Implantable Cardioverter Defibrillator; IQR=interquartile range; LBBB=Left Bundle Branch Block; LGE=Late Gadolinium Enhancement; LV=Left Ventricular; LVDD=Left Ventricular Diastolic Diameter; LV = Left Ventricle; NSVT= Non-sustained Ventricular Tachycardia; P/LP= Pathogenetic/Likely Pathogenetic; PVC=Premature Ventricular Contractions; RV=Right Ventricle;

|  | **No**  **LV systolic disfunction at baseline**  **(n=224)** | **No**  **LV systolic disfunction at follow up**  **(n=143, 64%)** | **LV systolic disfunction at follow up**  **(n=81, 36%)** |
| --- | --- | --- | --- |
| Age at visit (years, IQR) | 31 (21-44) | 30 (18-42) | 31 (23-44) |
| Sex (male) | 117 (52%) | 66 (46%) | 51 (63%) |
| Familial ALVC | 59 (26%) | 27 (19%) | 30 (37%) |
| Family history of SCD | 44 (20%) | 17 (12%) | 27 (33%) |
| History of Syncope | 14 (6%) | 6 (4%) | 8 (10%) |
| P/LP variant | 99 (44%) | 53 (37%) | 46 (57%) |
| Arrhythmogenic genes* | 36 (17%) | 15 (11%) | 21 (26%) |
| Arterial Hypertension | 31 (14%) | 15 (11%) | 16 (20%) |
| Hypercholesterolemia | 33 (15%) | 16 (11%) | 17 (21%) |
| Diabetes Mellitus | 7 (3%) | 3 (2%) | 4 (5%) |
| Smoke | 45 (20%) | 23 (16%) | 22 (27%) |
| Alcohol assumption | 4 (2%) | 2 (1%) | 2 (2%) |
| Sports (Competitive or High-intensity recreational Exercise) | 66 (30%) | 39 (27%) | 27 (33%) |
| Coronary artery disease | 2 (1%) | 1 (1%) | 0 |
| Previous myocarditis | 4 (2%) | 3 (2%) | 1 (1%) |
| Chemotherapy | 1 (0.4%) | 1 (1%) | 0 |
| Pregnancy  (Total number) | 21 (19%) (108) | 12 (15%) (78) | 9 (30%) (30) |

**Supplementary Table 7. Baseline risk factors for LV systolic disfunction development**. The major risk factors for development of LV systolic disfunction are the history of familial ALVC, being carrier of a P/LP variant and family history of SCD.

*Arrhythmogenic genes considered are LMNA, FLNC, TMEM43, PLN, DSP, RMB20, PKP2 and SCN5A.(6)

DCM=Dilated Cardiomyopathy; DSP=Desmoplakin; FLNC= filamin C; LMNA=lamin A; LV = Left Ventricle; PLN=Phospholamban; PKP2=Plakophilin-2; P/LP= Pathogenetic/Likely Pathogenetic; RBM20= RNA Binding Motif Protein 20; SCD=Sudden Cardiac Death; SCN5A= Sodium Voltage-Gated Channel Alpha Subunit 5; TMEM43= Transmembrane Protein 43.

|  | **No LV systolic disfunction**  **at baseline**  **(n=224)** | **No LV systolic disfunction**  **at follow-up**  **(n=143, 64%)** | **LV systolic disfunction**  **at follow-up**  **(n=81, 36%)** |
| --- | --- | --- | --- |
| **ECG at baseline** | | | |
| Pathologic ECG (%) | 35 (16%) | 8 (6%) | 27 (33%) |
| Sinus Rhythm (%) | 141 (98%) | 141 (99%) | 79 (97%) |
| QRS (ms) | 98±16 | 95±16 | 102±14 |
| PQ (ms) | 155±30 | 151±23 | 168±43 |
| 1^st^ degree AV block (%) | 10 (4%) | 1 (0.7%) | 9 (11%) |
| LAFB (%) | 6 (3%) | 2 (1%) | 4 (5%) |
| LBBB (%) | 8 (4%) | 0 (0%) | 8 (10%) |
| RBBB (%) | 9 (4%) | 8 (6%) | 1 (1%) |
| Negative T waves | 23 (10%) | 6 (4%) | 17 (21%) |
| Q waves | 4 (2%) | 0 (0%) | 4 (5%) |
| Fragmented QRS | 7 (3%) | 0 (0%) | 7 (9%) |
| Low voltages | 6 (3%) | 0 (0%) | 6 (7%) |
| LV hypertrophy | 6 (3%) | 2 (1%) | 4 (5%) |
| **ECG Holter at baseline** | | | |
| Pathological ECG Holter (%)  (Missing) | 60 (27%)  49 (22%) | 8 (8%)  38 (27%) | 50 (71%)  11 (14%) |
| PVC≥1000/24h (%)  (Missing) | 43 (25%)  49 (22%) | 2 (2%)  38 (27%) | 41 (59%)  11 (14%) |
| NSVT > 3 beats  (Missing) | 30 (17%)  49 (22%) | 1 (1%)  38 (27%) | 29 (41%)  11 (14%) |
| High-grade AV blocks (%)  (Missing) | 4 (2%)  49 (22%) | 6 (6%)  38 (27%) | 6 (9%)  11 (14%) |
| AF (%)  (Missing) | 12 (7%)  49 (22%) | 0 (0%)  38 (27%) | 4 (6%)  11 (14%) |
| PVC≥500/24h (%)  (Missing) | 58 (33%)  49 (22%) | 4 (4%)  38 (27%) | 54 (77%)  11 (14%) |
| PVC couplets≥50/24h  (Missing) | 9 (5%)  49 (22%) | 1 (1%)  38 (27%) | 54 (10%)  11 (14%) |
| **Echocardiography at baseline** | | | |
| Echo/abnormalities | 43 (19%) | 13 (9%) | 30 (31%) |
| LV dilatation (%) | 24 (11%) | 7 (5%) | 17 (21%) |
| LVEDD (mm) | 50 (44-57) | 48 (42-55) | 56 (46-60) |
| LVEF (%) | 59±6 | 61 (57-65) | 53 (51-58) |
| Abnormal segmental kinetics LV (%) | 32 (14%) | 7 (5%) | 25 (31%) |
| LV GLS (%)  Missing (%) | -18.0 ±4.0  20 (10%) | -19.1±1.9  14 (10%) | -15.6±3.5  6 (7%) |
| LV GLS < 18% (%) | 85 (42%) | 21 (16%) | 64 (85%) |
| Diastolic Dysfunction (grade II-III) (%) | 11 (5%) | 7 (3%) | 4 (5%) |
| Abnormal segmental kinetics RV (%) | 7 (3%) | 1 (0.7%) | 6 (7%) |
| Mitral regurgitation (moderate-severe) | 5 (2%) | 1 (0.7%) | 4 (5%) |
| Mitral Prolapse (%) | 11 (5%) | 4 (3%) | 7 (9%) |
| Tricuspid regurgitation (%) | 71 (32%) | 54 (38%) | 17 (21%) |
| **CMR at baseline** | | | |
| LGE (%)  Missing | 16 (23%)  153 (69%) | 3 (7%)  102 (72%) | 13 (43%)  51 (63%) |

**Supplementary Table 8. Clinical Predictors of Disease**. At baseline, relatives that develop LV systolic disfunction have more frequently abnormal signs at ECG and ECG-Holter than unaffected relatives. Furthermore, they show more remodeled hearts at Echocardiograms and high amounts of fibrosis at CMR. AF=Atrial Fibrillation; AV=Atrio-Ventricular; CMR=Cardiac Magnetic Resonance; EF= Ejection Fraction; GLS= Global Longitudinal Strain; LAFB=Left Anterior Fascicular Block; LBBB=Left Bundle Branch Block; LGE=Late Gadolinium Enhancement; LV=Left Ventricle; LVEDD=Left Ventricular End-Diastolic Diameter; NSVT= Non-sustained Ventricular Tachycardia; PVC=Premature Ventricular Contractions; RBBB=Right Bundle Branch Block; RV=Right Ventricle.

|  | **Hazard Ratio** | **95% Confidence Interval** | **P-value** |
| --- | --- | --- | --- |
| **Model 1 (ECG+Echo)** | | | |
| ECG Multivariable | 1.45 | 1.18-3.65 | **0.001** |
| Echocardiogram Multivariable | 2.43 | 1.44-4.10 | **<0.001** |
| **Model 2 (ECG+Echo+ECG Holter)** | | | |
| ECG Multivariable | 0.88 | 0.48-1.67 | 0.7 |
| Echocardiogram Multivariable | 1.69 | 0.97-2.93 | 0.063 |
| ECG-Holter Multivariable | 5.52 | 3.02-10.16 | **<0.001** |
| **Model 3 (ECG+Echo+ECG-Holter+LV-GLS)** | | | |
| ECG Multivariable | 0.72 | 0.38-1.33 | 0.6 |
| Echocardiogram Multivariable | 1.59 | 0.92-2.74 | 0.094 |
| ECG-Holter Multivariable | 4.02 | 2.07-7.79 | **<0.001** |
| LV-GLS Multivariable | 1.11 | 1.02-1.20 | **0.011** |

**Supplementary Table 9. Multivariable models for prediction of LV systolic disfunction in unaffected relatives at the baseline.** Comparison of multivariable models of ECG, Echocardiogram, ECG-Holter and GLS. *All the 3 model are corrected for the age at first visit and sex and including only subjects with all available data (156 subjects out of the 224).

GLS=Global Longitudinal Strain; LV=Left Ventricular.


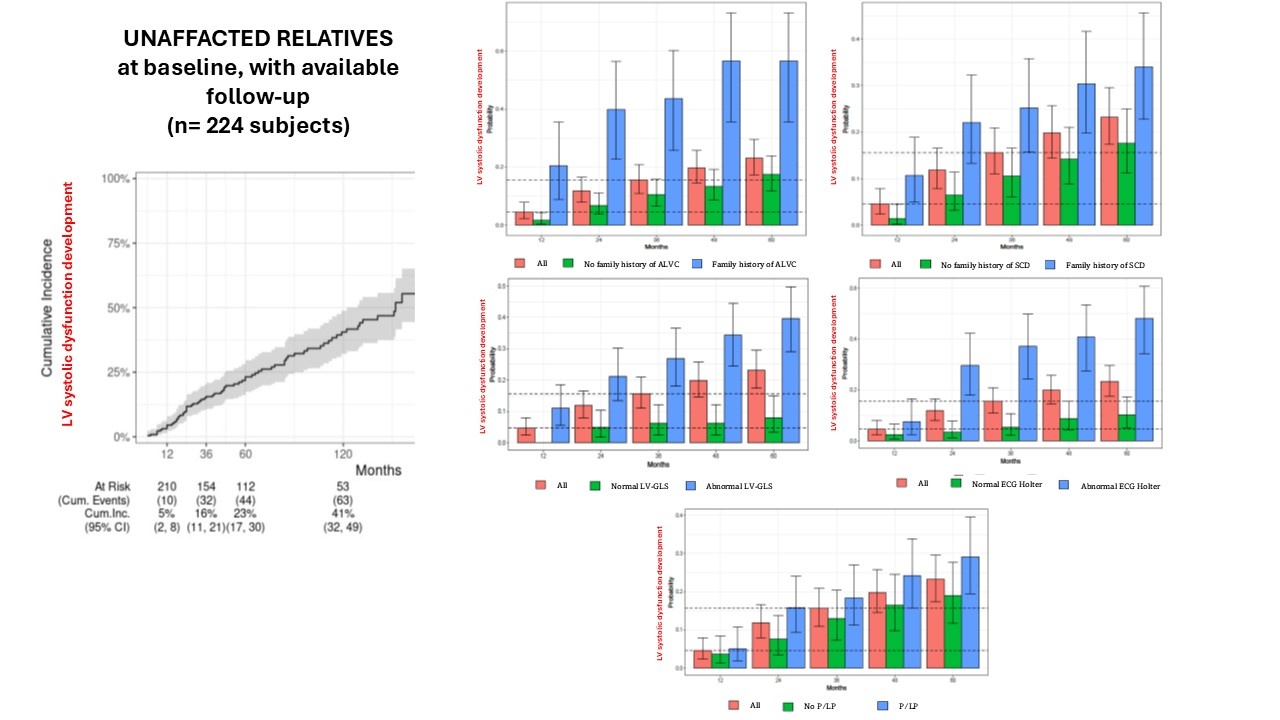


**Supplementary Figure 1 . Risk of LV systolic disfunction development for year of follow-up.**

Cumulative incidence of LV systolic disfunction for the total study population unaffected at the baseline and in presence of each single risk factor.

The dotted black line indicates the 1-to-3-year screening risk in the overall at-risk population. Error bars indicate 95% CI.

LV= left ventricle; ALVC= arrhythmogenic left ventricle cardiomyopathy; P/LP= Pathogenetic/Likely Pathogenetic; GLS=Global Longitudinal Strain; SCD= sudden cardiac death


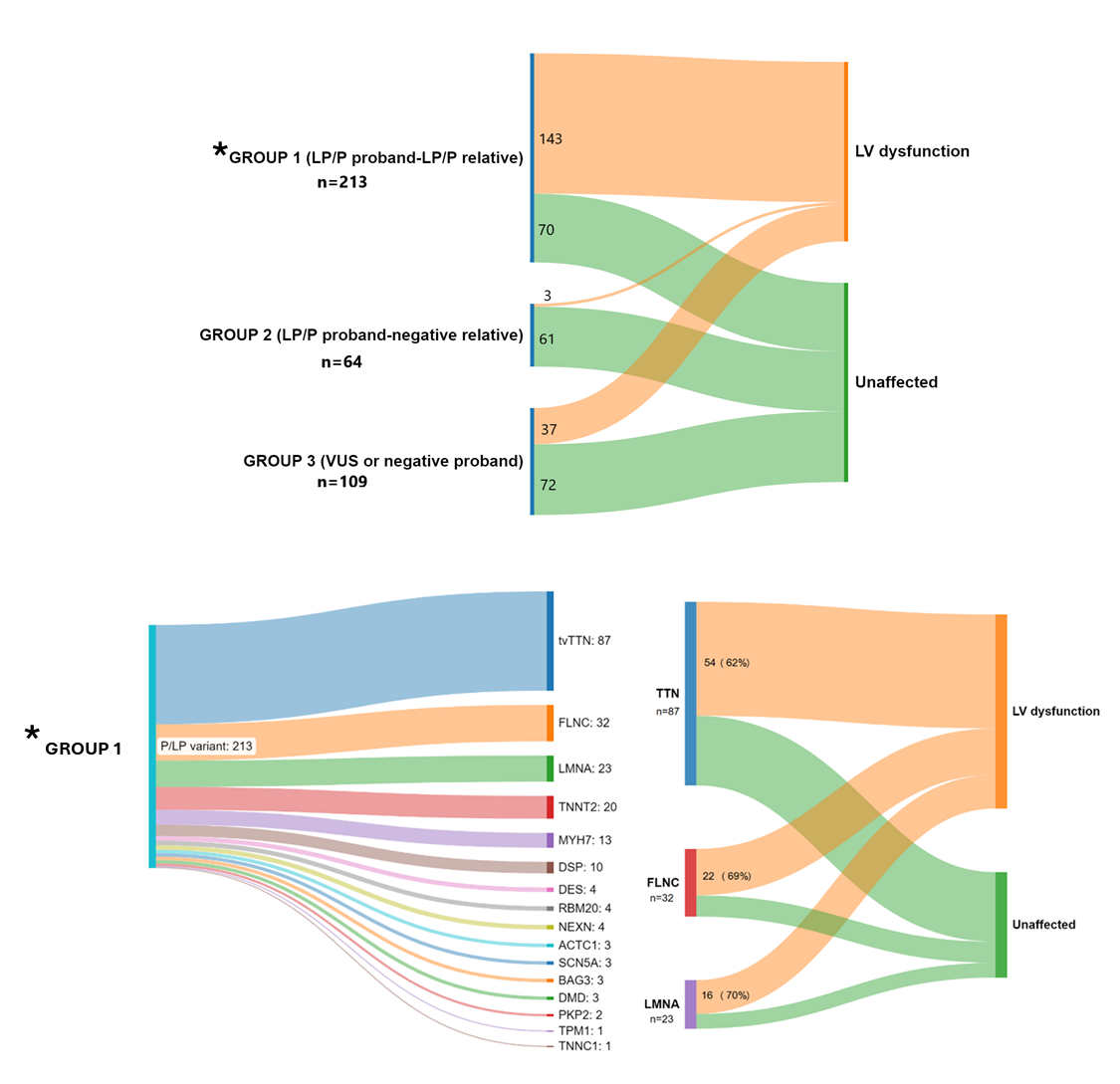


**Supplementary Figure 2. Sankey Diagrams for genetic characterization of relatives and development of LV systolic disfunction.**

Group 1: P/LP variant in proband, P/LP variant in relative; Group 2: P/LP variant in proband, negative relative; Group 3: VUS variant or negative result in proband.
The main genes involved in P/LP variants are TTN (40%), FLNC (15%) and LMNA (11%). Relatives that are carriers of P/LP variants in genes of TTN, FLNC and LMNA developed LV systolic dysfunction in 62%, 69% and 70% of cases, respectively. FLNC and LMNA confirm their high penetrance.
FLNC=Filamin C; LMNA= Lamin A; LV=Left Ventricular; P/LP= Pathogenetic/Likely Pathogenetic; TTN= Titin; LV=Left Ventricle; P/LP= Pathogenetic/Likely Pathogenetic; VUS=Variant of Uncertain Significance.





**Supplementary Figure 3. Cubic spline for LV-GLS. LV-GLS cut-off prediction for cardiac events in all family members independently of the clinical status at baseline**. The line represents the hazard ratio. The gray area represents the 95% confidence interval. The dashed line represents the hazard ratio of 1.
*All individuals that experienced primary outcome and secondary outcomes had mostly a LV-GLS minor than -17%.

GLS=Global Longitudinal Strain; HF=Heart Failure; LV=Left Ventricle.





**Supplementary Figure 4. Cubic spline for LV-GLS. LV-GLS cut-off prediction for cardiac events in family members with LV systolic disfunction already at baseline**. The line represents the hazard ratio. The gray area represents the 95% confidence interval. The dashed line represents the hazard ratio of 1.
*All individuals that experienced primary outcome and secondary outcomes in this subgroup of relatives, had mostly a LV-GLS minor than LV-GLS -13%.

GLS=Global Longitudinal Strain; HF=Heart Failure; LV=Left Ventricle.

**
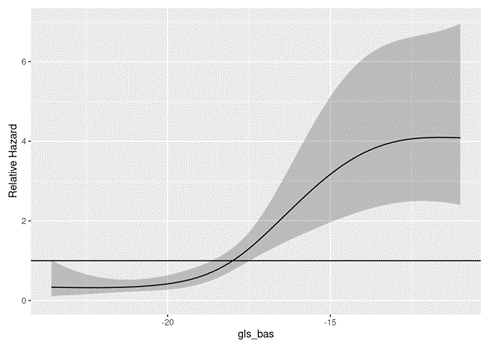
**

**Supplementary Figure 5**. **Cubic spline for LV-GLS**. **LV-GLS cut-off prediction for LV systolic disfunction in relatives without LV systolic disfunction at the baseline.** The line represents the hazard ratio. The gray area represents the 95% confidence interval. The dashed line represents the hazard ratio of 1. Based on the cubic spline analysis, -18% of LV-GLS appeared as an appropriate cut-off for dichotomization. The analysis including LV-GLS as a dichotomous variable, confirmed all results.

GLS=Global Longitudinal Strain; LV=Left Ventricle.

**REFERENCES**

1. Pinto YM, Elliott PM, Arbustini E, Adler Y, Anastasakis A, Böhm M, et al. Proposal for a revised definition of dilated cardiomyopathy, hypokinetic non-dilated cardiomyopathy, and its implications for clinical practice: a position statement of the ESC working group on myocardial and pericardial diseases. Eur Heart J. 2016 Jun 14;37(23):1850–8.

2. Finocchiaro G, Sheikh N, Biagini E, Papadakis M, Maurizi N, Sinagra G, et al. The electrocardiogram in the diagnosis and management of patients with hypertrophic cardiomyopathy. Heart Rhythm. 2020 Jan;17(1):142–51.

3. Taylor MRG, Carniel E, Mestroni L. Cardiomyopathy, familial dilated. Orphanet J Rare Dis. 2006 Jul 13;1:27.

4. Lang RM, Badano LP, Mor-Avi V, Aﬁlalo J, Armstrong A, Ernande L, et al. Recommendations for Cardiac Chamber Quantification by Echocardiography in Adults: An Update from the American Society of Echocardiography and the European Association of Cardiovascular Imaging. Journal of the American Society of Echocardiography. 2015;28(1).

5. Paldino A, De Angelis G, Dal Ferro M, Faganello G, Porcari A, Barbati G, et al. High prevalence of subtle systolic and diastolic dysfunction in genotype-positive phenotype-negative relatives of dilated cardiomyopathy patients. Int J Cardiol. 2021 Feb 1;324:108–14.

6. Arbelo E, Protonotarios A, Gimeno JR, Arbustini E, Barriales-Villa R, Basso C, et al. 2023 ESC Guidelines for the management of cardiomyopathies. Eur Heart J. 2023 Oct 1;44(37):3503–626.
